# Dietary macronutrient intake according to sex and trait anxiety level among non-diabetic adults: a cross-sectional study

**DOI:** 10.1186/s12937-021-00733-1

**Published:** 2021-09-08

**Authors:** Junko Kose, Léopold K. Fezeu, Mathilde Touvier, Sandrine Péneau, Serge Hercberg, Pilar Galan, Valentina A. Andreeva

**Affiliations:** 1 Nutritional Epidemiology Research Group (EREN), Sorbonne Paris Nord University, INSERM U1153/INRAE U1125/CNAM, Epidemiology and Statistics Research Center (CRESS) – University of Paris, 74 Rue Marcel Cachin, 93017 Bobigny, France; 2 Department of Public Health, AP-HP Avicenne Hospital, Bobigny, France

**Keywords:** Dietary intake, Macronutrients, Carbohydrates, Protein, Fat, Anxiety, Mental health, Epidemiological study

## Abstract

**Background:**

Studies suggest that anxiety is correlated with eating behavior, however, little is known about the association between anxiety status as predictor of dietary macronutrient intake. The aim of the present study was to investigate the sex-stratified cross-sectional associations of trait anxiety with intake of various macronutrients in a large population-based sample of non-diabetic adults.

**Methods:**

*N* = 20,231 participants (mean age = 53.7 ± 13.6 years) of the NutriNet-Santé web-cohort, who had completed the trait anxiety subscale of the Spielberger State-Trait Anxiety Inventory (T-STAI; 2013–2016) were included in the analyses. Dietary intake was calculated from at least 3 self-administered 24-h dietary records. The associations of interest were assessed by multiple linear regression stratified by sex, owing to significant interaction tests.

**Results:**

In total, 74.3% (*n* = 15,033) of the sample were females who had a significantly higher mean T-STAI score than did males (39.0 versus 34.8; *p* < 0.01). Among females, the fully-adjusted analyses showed significant positive associations of T-STAI with total carbohydrate intake (β = 0.04; *p* < 0.04), complex carbohydrate intake (β = 0.05; *p* < 0.02), and percentage energy from carbohydrates (β = 0.01; *p* < 0.03), as well as a significant inverse association of T-STAI with percentage energy from fat (β = -0.01; *p* < 0.05). As regards males, the only significant finding was an inverse association between T-STAI and percent of the mean daily energy from protein (fully-adjusted model: β = -0.01; *p* = 0.05).

**Conclusion:**

This cross-sectional study found modest sex-specific associations between anxiety status and macronutrient intake among French non-diabetic adults. Prospective studies are needed to further elucidate the observed associations.

## Introduction

In 2016, mental and addictive disorders affected more than 13% of the world’s population. Specifically, anxiety disorders, which are among the most common mental disorders and are associated with high individual-level health care costs [1], represent a substantial and growing proportion in the global disease burden. For example, the number of disability-adjusted life years attributable to anxiety disorders increased by 53.7% from 1990 to 2019 in all ages [2]. In addition, studies reveal high comorbidity of anxiety disorders with other mental disorders [3], physical disorders [4], and poor quality of life [5]. Hence, anxiety disorders—as an exposure and an outcome—merit increased attention by epidemiological and prevention research.

During the last decade, cross-sectional studies in the field of nutritional psychiatry have reported significant associations of anxiety disorders with dietary patterns/quality [6], intake of food groups [7] and certain micronutrients [8]. Plausible mechanisms pertain to the impact of dietary intake on gut microbiome composition, inflammation, and immune system capacity, each of which had an association with mental illness [9]. It has also been reported that mood could influence food intake. Moreover, studies have shown that males were more likely to report a positive emotional state before eating palatable foods, while negative emotions triggered the same behavior in females [10]. A study with 26 pairs of twins, investigating the link between anxiety and ad libitum food intake, found that anxiety status had a sex-specific influence on caloric and macronutrient intake [11]. To our knowledge, no large population-based study has investigated dietary intake according to anxiety status. Existing epidemiological studies regarding the impact of dietary intake on anxiety status often focus on certain food groups or micronutrients and relatively small sample sizes [6–8]. As a result, little is known at present about the association between anxiety status and intake of dietary macronutrients in the general population.

The main objective of the present study was to assess the association of trait anxiety with intake of various macronutrients in a large population-based adult sample. Given differences between males and females in the prevalence of anxiety disorders [3] and also in dietary intake [12], our secondary objective was to assess whether the association between trait anxiety and macronutrient intake varied by sex.

## Subjects and methods

### The NutriNet-Santé study

The nationwide NutriNet-Santé study was launched in France 12 years ago (in May 2009) and is still ongoing. It is a large-scale, prospective web-cohort intended to provide evidence about the direct and indirect relationship between nutrition and physical and mental health. Details about the study design and protocol are available elsewhere [13]. Briefly, adults aged 18 years and older with Internet access are recruited from the general population via multiple traditional (e.g., flyers in doctors’ offices, media campaigns) and online (e.g., website ads) strategies. At inclusion and following the provision of an electronic informed consent, participants complete a set of five principal questionnaires (repeated on a biannual and/or annual basis thereafter) related to diet, physical and mental health status, anthropometrics, physical activity, and socio-demographic and lifestyle characteristics (described below).

The NutriNet-Santé study is conducted according to the Declaration of Helsinki guidelines. It was approved by the Institutional Review Board of the French Institute for Health and Medical Research (INSERM # 00000388FWA00005831) and by the National Commission on Informatics and Liberty (CNIL # 908450 and # 909216). NutriNet-Santé is registered (# NCT03335644) at www.ClinicalTrials.gov.

## Measures

### Dietary intake

Macronutrient intake was the outcome of interest in this analysis. In the NutriNet-Santé study, dietary intake is evaluated at inclusion and every 6 months thereafter, each time using three non-consecutive 24-h dietary records. The dietary data collection tool has been validated against dietitian interviews and against nutritional status biomarkers [14, 15]. For each diet assessment day, participants were asked to report all food, beverages, and composite dishes consumed, along with the portion size/quantity, the recipe and/or seasoning for each item, and the meal setting (place, time, company, etc.). Portion sizes were recorded with the help of validated photographs [16], standard serving containers or directly in *g* or *ml*. NutriNet-Santé has its own food composition table that includes over 3,500 different items; it was used to calculate mean daily energy and macronutrient intake [17]. All reported dietary data were weighted in order to respect the 5:7 and 2:7 ratios of week days and weekend days. Potential dietary energy under-reporting was identified by applying the Goldberg cut-off [18], taking into account the individual’s age, sex, weight, height, physical activity level, and basal metabolic rate. In the present study, each participant’s macronutrient intake was averaged across a minimum of three 24-h dietary records provided within a 2.5-year period around the anxiety questionnaire completion date (described below). Individuals flagged for energy under-reporting were excluded from the analysis in order to strengthen the validity of dietary data. Likewise, individuals with prevalent or incident diabetes mellitus (type 1 or type 2) and females who were pregnant at the time of dietary intake assessment were ineligible for this study owing to potential specificities of their dietary regimens.

Overall, several outcome variables related to macronutrient intake were modelled: mean total carbohydrates (g/d), mean complex carbohydrates (g/d), mean simple sugars (g/d), mean protein (g/d), mean fat (g/d), mean monounsaturated fatty acids (MUFA) (g/d), mean polyunsaturated fatty acids (PUFA) (g/d), mean saturated fatty acids (SFA) (g/d), and percentage energy in the total diet from carbohydrates, protein and fat, respectively. Nutrient intake was energy-adjusted using the residual method [19].

### Trait anxiety

For each participant, anxiety status—which was the main independent variable in this analysis—was assessed once during the 2013–2016 period (*N* = 119,451 solicited participants). Specifically, proneness to anxiety was evaluated by means of self-reports on the State-Trait Anxiety Inventory Form Y (STAI) which is one of the most widely used epidemiological tools for evaluating general anxiety proneness, distinguishing it from depression [20]. The French version of STAI has been validated in adults from the general population [21]. In line with the objectives of the study, and consistent with prior research [22], only the trait-anxiety subscale (T-STAI), which assesses a relatively stable personal characteristic, was used for the analysis. T-STAI contains 20 items scored on a 4-point Likert scale (with reverse-scoring for some items) ranging from “Almost never” to “Almost always” (minimum 20 points, maximum 80 points). Examples of questions include: “I worry too much over something that really doesn’t matter” and “I am a steady person.” The higher the score, the greater one’s proneness to anxiety. Trait anxiety measured by T-STAI was reported to be highly correlated with generalized anxiety disorder [23].

### Covariates

Self-reported data on age, sex, educational level, socio-professional category, marital status, alcohol consumption, and smoking status were collected by a previously validated questionnaire [24]. Physical activity was assessed by the validated International Physical Activity Questionnaire and scoring was based on established criteria [25]. Height and weight were self-reported using a previously validated anthropometrics questionnaire [26]. Information about prescription medication use for various mental and/or psycho-neurological conditions (memory impairment, Alzheimer’s disease, anorexia nervosa, bipolar disorder, migraine, major depression, epilepsy, neuralgia, Parkinson’s disease, sleep disorders, etc.) was collected using the health status questionnaire. Similar to the dietary data approach, we relied on covariate data provided over a 2.5-year period around the T-STAI completion date. Finally, the number of available 24-h dietary records (with the minimum set at 3) was also modelled as a covariate.

### Statistical analysis

Body mass index (BMI, kg/m^2^) was calculated based on the self-reported height and weight data. Next, for individuals lacking information about any of the covariates, two approaches were used: if, in the full sample, more than 5% of the data for any given covariate were missing, then a “not reported” category was created for each variable with missing values in order to maintain these participants in the sample; if missing values pertained to less than 5% in the full sample, those participants were excluded from the analysis. As regards the variable “socio-professional category,” whenever the value was missing and age was < 25 or > 60 years, the respective status of “student” and “retired” was attributed. Descriptive characteristics by sex are presented in Table 1 and reflect number (percent) from chi-squared tests for categorical variables and mean (± SD) values from Student *t* tests for continuous variables. In the linear regression analyses, the main independent variable was trait anxiety (modelled on a continuous scale) and the dependent variables pertained to energy-adjusted mean macronutrient intake (each modelled on a continuous scale). Model 1 was adjusted for age (years, continuous scale). Model 2 was adjusted for age (years, continuous scale), BMI (kg/m^2^, continuous scale), alcohol consumption (g ethanol/d, continuous scale), smoking status (never, former, current smoker), physical activity level (low, moderate, high, not reported), educational level (less than high school, high school diploma or equivalent, college/undergraduate degree, graduate degree, not reported), socio-professional category (homemaker/disabled/unemployed/student, manual/blue collar/office work/administrative staff, professional/executive staff, retired), marital status (living alone or married/cohabiting), and number of 24-h dietary records (continuous scale). Finally, a sensitivity analysis (Model 3) took into account prescription medication use for mental illness (yes or no) in addition to the covariates included in Model 2. Tests for interaction (significance level *p* < 0.10) by age, sex, BMI, and smoking status were also performed. The main tests were two-sided and *p* < 0.05 was considered as evidence for statistical significance. SAS® version 9.4 (SAS Institute, Inc., Cary NC, USA) was used for all statistical analyses.

**Table 1:** Descriptive characteristics of NutriNet-Santé participants according to sex (*N* = 20,231)

	**Full sample**	**Males**	**Females**	***P*-value**^**1**^
	***N* = 20,231**	***n* = 5,198**	***n* = 15,033**	
**T-STAI score**^**a**^**, mean (SD)**	37.92 (10.03)	34.83 (9.30)	38.98 (10.05)	< *0.0001*
**Age, years, mean (SD)**	53.68 (13.63)	58.48 (13.07)	52.02 (13.43)	< *0.0001*
**Age category**				
18–34 y	2,358 (11.66)	312 (6.00)	2,046 (13.61)	< *0.0001*
35–54 y	7,109 (35.14)	1,406 (27.05)	5,703 (37.94)	
55–64 y	5,472 (27.05)	1,286 (24.74)	4,186 (27.85)	
≥ 65 y	5,292 (26.16)	2,194 (42.21)	3,098 (20.61)	
**Educational level**				
Less than high school	2,908 (14.37)	916 (17.62)	1,992 (13.25)	< *0.0001*
High school diploma or equivalent	3,586 (17.73)	958 (18.43)	2,628 (17.48)	
College, under graduate degree	5,461 (26.99)	1,126 (21.66)	4,335 (28.84)	
Graduate degree	6,988 (34.54)	2,049 (39.42)	4,939 (32.85)	
Not reported	1,288 (6.37)	149 (2.87)	1,139 (7.58)	
**Socio-professional category**				
Homemaker/ disabled/ unemployed/ student/ trainee	2,255 (11.15)	236 (4.54)	2,019 (13.43)	< *0.0001*
Manual/ blue collar/ office work/ administrative staff	6,726 (33.25)	1,055 (20.30)	5,671 (37.72)	
Professional/ executive staff	4,714 (23.30)	1,372 (26.39)	3,342 (22.23)	
Retired	6,536 (32.31)	2,535 (48.77)	4,001 (26.61)	
**Marital status**				
Living alone (single, divorced, widowed)	4,827 (23.86)	881 (16.95)	3,946 (26.25)	< *0.0001*
Married/ cohabiting	15,404 (76.14)	4,317 (83.05)	11,087 (73.75)	
**Physical activity**^**b**^				
Low	3,588 (17.74)	824 (15.85)	2,764 (18.39)	< *0.0001*
Moderate	7,541 (37.27)	1,734 (33.36)	5,807 (38.63)	
High	6,589 (32.57)	2,117 (40.73)	4,472 (29.75)	
Not reported	2,513 (12.42)	523 (10.06)	1,990 (13.24)	
**Body Mass Index (BMI, kg/m2), mean (SD)**	23.59 (3.86)	24.72 (3.21)	23.20 (3.99)	< *0.0001*
**BMI category**				
Underweight (< 18.5)	917 (4.53)	47 (0.90)	870 (5.79)	< *0.0001*
Normal weight (18.5–24.9)	13,401 (66.24)	2,999 (57.70)	10,402 (69.19)	
Overweight (25.0–29.9)	4,693 (23.20)	1,854 (35.67)	2,839 (18.89)	
Obese (≥ 30)	1,220 (6.03)	298 (5.73)	922 (6.13)	
**Smoking status**				
Never smoker	10,234 (50.59)	2,190 (42.13)	8,044 (53.51)	< *0.0001*
Former smoker	7,867 (38.89)	2,505 (48.19)	5,362 (35.67)	
Current smoker	2,130 (10.53)	503 (9.68)	1,627 (10.82)	
**Medication for mental illness**^**c**^				
Yes	6,939 (34.30)	1,132 (21.78)	5,807 (38.63)	< *0.0001*
No	13,292 (65.70)	4,066 (78.22)	9,226 (61.37)	
**Alcohol use, g ethanol/d, mean (SD)**	8.48 (11.48)	15.12 (16.03)	6.18 (8.25)	< *0.0001*
**Total energy intake, Kcal/d, mean (SD)**	1,910.46 (441.47)	2,284.76 (454.38)	1,781.03 (354.55)	< *0.0001*
**Number of 24-h dietary records, mean (SD)**	7.01 (2.84)	7.39 (2.83)	6.89 (2.83)	< *0.0001*

Values refer to number (%) except when noted otherwise. Values are rounded off to two decimal places

^1^*P*-values obtained from chi-squared tests or Student *t* tests, as appropriate

^a^Spielberger Trait Anxiety Inventory (T-STAI), form Y; score range between 20 and 80 points, with higher scores reflecting higher proneness to anxiety

^b^Assessed with the International Physical Activity Questionnaire-Short Form according to established scoring criteria

^c^At least one disorder among the following: addiction, Alzheimer’s disease, anorexia nervosa, bipolar disorder, non-migraine headache, migraine, major depression, epilepsy, memory impairment, neuralgia, Parkinson’s disease, sleep disorders, or other mental health condition

## Results

### Description of the participants

**Fig. 1:**
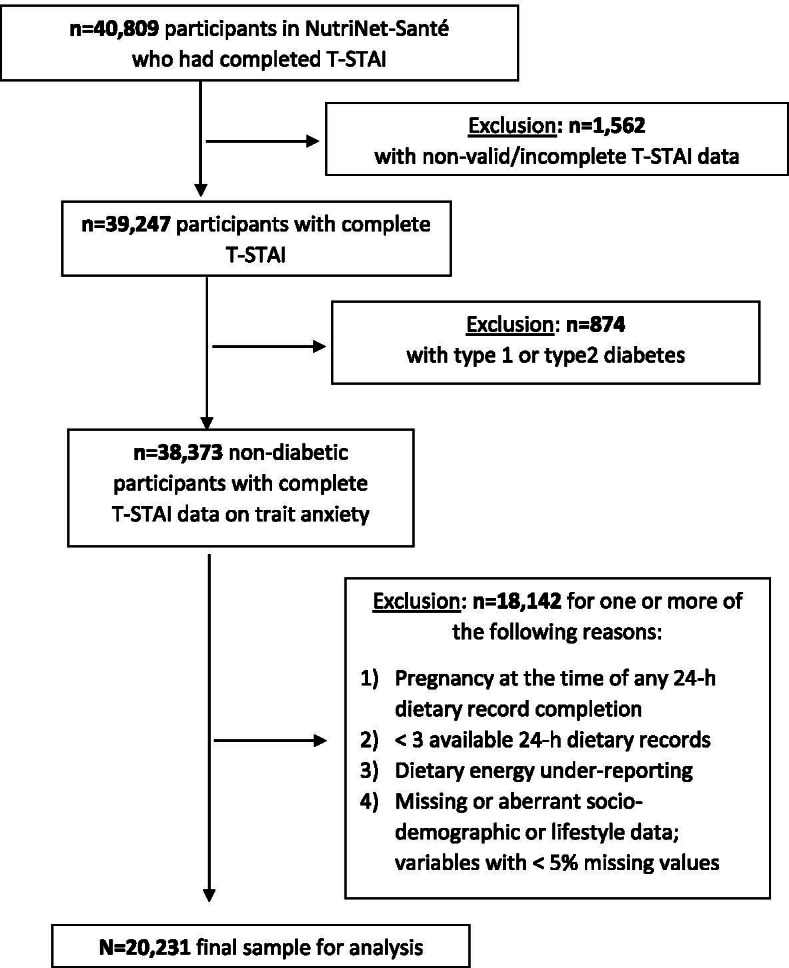
Participant selection flowchart

In total, 40,809 NutriNet-Santé participants completed the T-STAI, of whom 1,562 had some non-valid, missing, or partial data and were excluded from the analyses. Next, those with prevalent or incident type 1 or type 2 diabetes were ineligible for the study (*n* = 874). Finally, a total of 18,142 individuals were excluded from the analysis sample owing to one or more of the following reasons: 1) pregnancy at the time of any 24-h dietary record completion, 2) fewer than three available 24-h dietary records, 3) dietary energy under-reporting, and 4) missing or incomplete socio-demographic and/or lifestyle data (< 5% missing values in the full sample). Thus, 20,231 participants (74.3% females; mean age = 53.7 ± 13.6 years) were included in the final sample for analysis (Fig. 1).

Descriptive characteristics according to sex are presented in Table 1. Females had significantly higher T-STAI score than did males (39.0 ± 10.1 versus 34.8 ± 9.3; *p* < 0.0001). They also had lower BMI, were somewhat younger, more likely to live alone, to be less physically active, non-smokers and to report prescription medication use compared to males (all *p* < 0.0001). Next, mean daily alcohol consumption and total daily calories were significantly lower among females than among males (all *p* < 0.0001). In the full sample, the mean number of 24-h dietary records was 7.0 ± 2.8.

### Association between trait anxiety and macronutrients intake

Unlike age, BMI, and smoking status, interaction by sex was statistically significant (*p* < 0.01). Hence, the principal analysis was stratified by sex. Table 2 (males) and Table 3 (females) present non-standardized beta coefficients regarding the association between T-STAI scores and macronutrient intake obtained from multiple linear regression models. Among females, significant positive associations with T-STAI score were observed for total carbohydrates (β = 0.04; *p* < 0.04), complex carbohydrate intake (β = 0.05; *p* < 0.02), and percent energy from carbohydrates (β = 0.01; *p* < 0.03) in Model 2. Furthermore, a significant inverse association between T-STAI and percent of mean daily energy from fat (β = -0.01; *p* < 0.05) emerged. As regards males, the only significant result from the fully-adjusted analysis (Model 2) pertained to an inverse association between T-STAI score and percent of total daily energy derived from protein (β = -0.01; *p* = 0.05). Also, a borderline significant inverse association for protein intake was found (β = -0.04; *p* < 0.08). Although the results were not significant, the observed beta coefficients for complex carbohydrates, simple sugars, and fat were similar in size to those found among females (β = 0.03, -0.04, and 0.02, respectively).

**Table 2:** Association between trait anxiety and mean macronutrient intake (g/day) among males in the NutriNet-Santé cohort (*n* = 5,198)

	Model 1		Model 2		Model 3 (Sensitivity analysis)	
	β	*p*	β	*p*	β	*p*
Total carbohydrates	-0,013	*0,82*	-0,010	*0,84*	-0,008	*0,88*
Complex carbohydrates	0,033	*0,47*	0,034	*0,44*	0,048	*0,29*
Simple sugars	-0,045	*0,30*	-0,043	*0,28*	-0,053	*0,19*
Protein	-0,035	*0,10*	-0,038	*0,07*	-0,031	*0,15*
Fat	0,035	*0,10*	0,024	*0,25*	0,021	*0,31*
MUFA	0,017	*0,12*	0,010	*0,36*	0,012	*0,27*
PUFA	0,006	*0,35*	0,004	*0,52*	0,001	*0,82*
SFA	0,012	*0,36*	0,010	*0,43*	0,008	*0,54*
Percentage energy from carbohydrates	-0,001	*0,90*	-0,001	*0,89*	-0,001	*0,93*
Percentage energy from fat	0,014	*0,10*	0,009	*0,24*	0,008	*0,34*
Percentage energy from protein	-0,007	*0,08*	-0,007	*0,05*	-0,006	*0,09*

Model 1: Multiple linear regression adjusted for age. Model 2: Multiple linear regression adjusted for age, BMI, alcohol consumption, smoking status, physical activity level, educational level, socio-professional category, marital status, and number of 24-h dietary records. Model 3, sensitivity analysis: Multiple linear regression adjusted for age, BMI, alcohol consumption, smoking status, physical activity level, educational level, socio-professional category, marital status, prescription medication use for mental illness, and number of 24-h dietary records. Values are rounded off to three and two decimal places for β coefficients and *p* values, respectively

*β* Non-standardized beta coefficients, *MUFA* Monounsaturated fatty acids, *PUFA* Polyunsaturated fatty acids, *SFA* Saturated fatty acids

**Table 3:** Association between trait anxiety and mean macronutrient intake (g/day) among females in the NutriNet-Santé cohort (*n* = 15,033)

	Model 1		Model 2		Model 3 (Sensitivity analysis)	
	β	*p*	β	*p*	β	*p*
Total carbohydrates	0,041	*0,07*	0,044	*0,03*	0,049	*0,02*
Complex carbohydrates	0,050	*0,01*	0,047	*0,01*	0,056	< *0,01*
Simple sugars	-0,009	*0,60*	-0,004	*0,82*	-0,008	*0,67*
Protein	-0,008	*0,43*	-0,011	*0,26*	-0,012	*0,22*
Fat	-0,001	*0,90*	-0,012	*0,16*	-0,014	*0,12*
MUFA	-0,004	*0,35*	-0,008	*0,11*	-0,008	*0,09*
PUFA	0,001	*0,73*	0,000	*0,86*	0,000	*0,92*
SFA	0,005	*0,37*	-0,001	*0,80*	-0,002	*0,65*
Percentage energy from carbohydrates	0,011	*0,02*	0,011	*0,02*	0,012	*0,01*
Percentage energy from fat	-0,004	*0,43*	-0,009	< *0,05*	-0,009	*0,04*
Percentage energy from protein	0,000	*0,92*	-0,001	*0,55*	-0,002	*0,45*

Model 1: Multiple linear regression adjusted for age. Model 2: Multiple linear regression adjusted for age, BMI, alcohol consumption, smoking status, physical activity level, educational level, socio-professional category, marital status, and number of 24-h dietary records. Model 3, sensitivity analysis: Multiple linear regression adjusted for age, BMI, mean alcohol consumption, smoking status, physical activity level, educational level, socio-professional category, marital status, prescription medication use for mental illness, and number of 24-h dietary records. Values are rounded off to three and two decimal places for β coefficients and *p* values, respectively

*β* Non-standardized beta coefficients, *MUFA* Monounsaturated fatty acids, *PUFA* Polyunsaturated fatty acids, *SFA* Saturated fatty acids

### Sensitivity analysis

In the sensitivity analysis (Model 3) where prescription medication use was added as a covariate in the models, the observed association between T-STAI and percentage from protein in males was attenuated and became non-significant. Compared to the main results, no other substantial change was observed among males or females. However, among females, beta coefficients for all macronutrients (except for PUFA) with significant results were somewhat higher in the sensitivity analysis than in the main analysis.

## Discussion

This epidemiological cross-sectional study, conducted in a large population-based sample of French non-diabetic adults, revealed modest sex-specific association between trait anxiety and macronutrient intake. Specifically, trait anxiety, which is regarded as a relatively stable personal characteristic and was modeled as the main exposure variable, was positively associated with intake of total carbohydrates (a one-point increase in T-STAI corresponded to an increase of 0.04 g in mean daily intake), complex carbohydrates (a one-point increase in T-STAI corresponded to 0.05 g increase in mean daily intake), and percentage of mean daily energy obtained from carbohydrates (a one-point increase in T-STAI corresponded to 0.01% increase in mean energy from carbohydrates) among females. In turn, null findings concerning carbohydrate intake were detected among males, in spite of the beta coefficients being similar in size to those found among females. Next, an inverse association was observed between trait anxiety and percent energy from fat among females (a one-point increase in T-STAI corresponded to 0.01% decrease in mean energy from fat). The respective associations among males were positive but did not reach statistical significance. Finally, regarding protein intake, there was a significant inverse association between trait anxiety and the percent energy from protein (a one-point increase in T-STAI corresponded to 0.01% decrease in mean energy from protein) among males, whereas null findings were observed among females. However, in the sensitivity analysis, where prescription medication use for mental disorders was added as a covariate in the analysis, the association of T-STAI with percentage energy from protein among males was attenuated, possibly due to insufficient statistical power.

Studies have reported that affect, stress and worry have a powerful influence on food choices, especially comfort/palatable food consumption, which could be considered as a compensatory behavior, because of its ability to enhance psychological well-being [10]. Interestingly, it has been reported that comfort/palatable food type preference differs according to sex: males tend to prefer meal-related foods such as steak or beef; females tend to prefer snack-related foods such as candy and chocolate [27]. Furthermore, significant sex differences in the emotional antecedents of comfort/palatable food consumption have been suggested. Males tended to report a positive emotional state before consuming palatable food, whereas the same behavior was triggered by negative affect in females [10]. These previous findings are consistent with the sex-specific associations observed in our study: carbohydrate intake in females was positively associated with trait anxiety, while there seemed to be an inverse association between protein intake and trait anxiety in males.

In turn, the underlying mechanisms of our findings may concern immune system/inflammation and neurotransmitter function, all of which have an impact on mental health status [28]. In addition, the association between anxiety and macronutrient intake may be bidirectional [9–11]. First, a diet with a high glycemic load was reported to be positively correlated with plasma concentrations of high-sensitivity C-reactive protein, an established marker of systemic inflammation [29]. The glycemic load reflects not only the quality (glycemic index), but also the quantity of carbohydrates in the diet. In our study, trait anxiety was positively correlated with total carbohydrate intake in females. As regards our variable for simple sugars, it consists of both sugars naturally present in food (e.g., fructose in fruits or lactose in dairy products) and added simple sugar. The intake of fruit and dairy products has been reported by some studies to have a protective effect on mental disorders [7, 30]. In contrast, studies suggest a strong association between added sugar consumption and mental disorder incidence [31]. The fact that the variable “simple sugar” in our study reflects both potential risk (i.e., added sugar intake) and protective factors (i.e., fresh fruit intake) may explain the null findings. Next*,* fat intake, seemed to be inversely correlated with trait anxiety in females. MUFA were previously reported to have inverse associations with depression [32], which displays a high level of comorbidity with anxiety disorders [33]. On the other hand, in the literature, omega-3 fatty acids (a type of PUFA) have been reported to have protective effects against anxiety [34]. No significant association was observed for PUFA in the present analysis, which may be partly explained by the fact that this variable in our database consisted of both omega-3 and omega-6 fatty acids. A clinical study had reported that blood levels of omega-3 fatty acids were significantly lower in comorbid depressive and anxiety disorder patients compared to healthy controls, whereas those of omega-6 fatty acids were not different between the two groups [35]. Finally, protein intake may have a beneficial effect on anxiety, since a major neurotransmitter, serotonin, is biosynthesized from the essential amino acid tryptophan. Serotonin has been advanced for nearly 30 years as a neurotransmitter influencing the expression of conditioned anxiety [36], and serotonin reuptake inhibitors are, to this day, used as the first-line pharmacological treatment of anxiety disorders [37].

In spite of the substantial burden of mental disorders including anxiety disorders and their comorbidity with other mental pathologies [3], mental health status is still not a public health priority [38]. Our results contribute to the accumulation of evidence to guide future research, intervention and public health policy efforts. It also becomes evident that the current Covid-19 pandemic is shifting attention towards mental illness [39, 40]. Given the high comorbidity of anxiety disorders with other mental conditions [3], future research could also investigate the relationship between comorbidity of mental disorders and dietary intake.

Several limitations of this study should be recognized. The cross- design prevents any inference of causality; it has been suggested that a bidirectional association might exist between dietary habits and mental health status [9–11]. Future prospective research is needed to shed light not only on causality but also on the potential bidirectional association between macronutrient intake and anxiety proneness. Next, our single time-point assessment of trait anxiety was based on a validated tool yet it does not correspond to any clinical diagnosis of anxiety disorders. However, the mean values of T-STAI score in the present study were consistent with previous studies using T-STAI [41]. Further, despite the statistical adjustment for a large number of pertinent covariables, residual confounding by unmeasured constructs (e.g., ethnoracial status, family history of anxiety disorders) might be present. Next, participation in the cohort is on voluntary basis; yet the widespread use of Internet in France (82% of French households had Internet access in 2013 when the STAI questionnaire was launched and > 90% had Internet access in 2019) is likely to have mitigated the selection bias to a certain extent [42]. Finally, as previously reported, NutriNet-Santé includes a higher proportion of females and individuals of high socio-economic status compared to the general French population [43], which bears on the external validity of the study. For instance, a high proportion of NutriNet-Santé participants (76.8% of females and 78.6% of males) appeared to meet recommended intake levels for sweet food [44]. In addition, because of the smaller number of male (versus female) participants, the available statistical power might have been impacted; yet the beta coefficients among males were of the same relative size as those observed among females.

Despite these limitations, the study presents several important strengths. To our knowledge, it is the first large epidemiological study to reveal associations between trait anxiety level as the exposure and macronutrient intake as the outcome. In addition, data were collected by validated instruments in a large and diverse sample of adults. Moreover, dietary macronutrient intake was estimated on the basis of a mean of seven 24-h dietary records previously validated against dietitian interviews and various biomarkers of nutritional status [14, 15].

## Conclusions

This cross-sectional study found modest sex-specific associations between trait anxiety and macronutrient intake in a large sample of non-diabetic adults recruited from the general population. Specifically, positive associations for carbohydrate intake and inverse associations for fat intake were observed in relation to trait anxiety among females, while protein intake was inversely associated with trait anxiety among males. In the future, prospective studies with representative samples and even randomized controlled trials featuring dietary interventions could advance knowledge about causality and the potential bidirectionality of the observed associations.

## Data Availability

Data used in this study are under the protection of national health data regulations set forth by the French National Commission on Informatics and Liberty (Commission Nationale de l’Informatique et des Libertés, CNIL) which prohibit unrestricted public access. The data can be made available upon written request sent to the NutriNet-Santé operational coordinator, Dr. Nathalie Druesne-Pecollo (n.pecollo@eren.smbh.univ-paris13.fr), and following approval by the NutriNet-Santé steering committee.

## Abbreviations

BMI: Body mass index
MUFA: Monounsaturated fatty acids
PUFA: Polyunsaturated fatty acids
SFA: Saturated fatty acids
T-STAI: Trait anxiety subscale of the Spielberger State-Trait Anxiety Inventory
